# Primary scrotal diffuse large B-cell lymphoma with skip metastasis to the mediastinum: a case report and literature review

**DOI:** 10.3389/fonc.2025.1698794

**Published:** 2025-12-09

**Authors:** Ziye Wang, Wen Tang, Daihui Xiao, Yi Li, Guobiao Liang, Zeju Zhao, Tao Wu

**Affiliations:** 1 Department of Urology, The Affiliated Hospital of Zunyi Medical University, Zunyi, China; 2 Department of Urology, Kaiyang County People’s Hospital, Kaiyang, Guizhou, China

**Keywords:** primary scrotal diffuse large B-cell lymphoma (PS-DLBCL), non-Hodgkin lymphoma (NHL), skip metastasis, B-cell, case report

## Abstract

Primary scrotal diffuse large B-cell lymphoma (PS-DLBCL) is an uncommon disease. Due to the scarcity of case reports, its atypical clinical manifestations often lead to misdiagnosis. An 81-year-old male patient presented with a rapidly enlarging, painless left scrotal mass. Imaging studies suggested sarcoma or seminoma. A chest computed tomography (CT) scan incidentally revealed mediastinal masses. Laboratory tests showed significantly elevated lactate dehydrogenase and β_2_-microglobulin levels. Postoperative histopathological examination confirmed DLBCL with a germinal center B-cell subtype, accompanied by an abnormally high Ki-67 proliferation index (>90%) and P53 translocation. FISH testing ruled out double-hit lymphoma. Notably, staging evaluation identified “isolated mediastinal involvement” without inguinal, pelvic, or retroperitoneal lymph node involvement, defining a “skip metastasis” pattern. The patient was diagnosed with stage IV disease and a high-risk International Prognostic Index (IPI) score of 5. Refusing chemotherapy, the patient died within 3 months of initial diagnosis due to rapid disease progression. This case highlights that scrotal lymphoma is rare yet highly aggressive, with clinical presentations frequently confused with common malignancies. Diagnosis relies on biopsy and immunohistochemistry, requiring clinicians to remain vigilant for early detection and intervention.

## Introduction

Diffuse large B-cell lymphoma (DLBCL) is the most common type of non-Hodgkin lymphoma (NHL) and is an aggressive disease that requires prompt treatment. It has unique morphological, immunohistochemical (IHC), or genetic characteristics and exceptional biological behavior (1). The usual clinical presentation is a rapid growth of the lymph node or extranodal mass. Among these, extranodal lymphoma accounts for 25%–35% of all NHL and can occur in almost any organ, with the most common extranodal site being the gastrointestinal tract (1). According to the relevant literature, most cases of urinary tract (UT) DLBCL originated from the kidney (72.39%), followed by the urinary bladder (24.95%) (2). Among them, the proportion of urogenital lymphoma is less than 5% (2). PS-DLBCL is a very rare extranodal lymphoma, and in pathological types, it is mostly DLBCL. Notably, in this case, the tumor completely bypassed the inguinal, pelvic, and retroperitoneal lymph nodes, resulting in an isolated mediastinal metastasis. This distinct “skip metastasis” pattern has not been previously reported in the literature on PS-DLBCL, underscoring its unique characteristics. Early diagnosis and systematized treatment are closely related to patient outcomes. This report describes a rare case of PS-DLBCL, highlighting the importance for clinicians to ensure early diagnosis and standardized and systematic treatment in the face of such diseases.

## Case presentation

**Figure 1:**
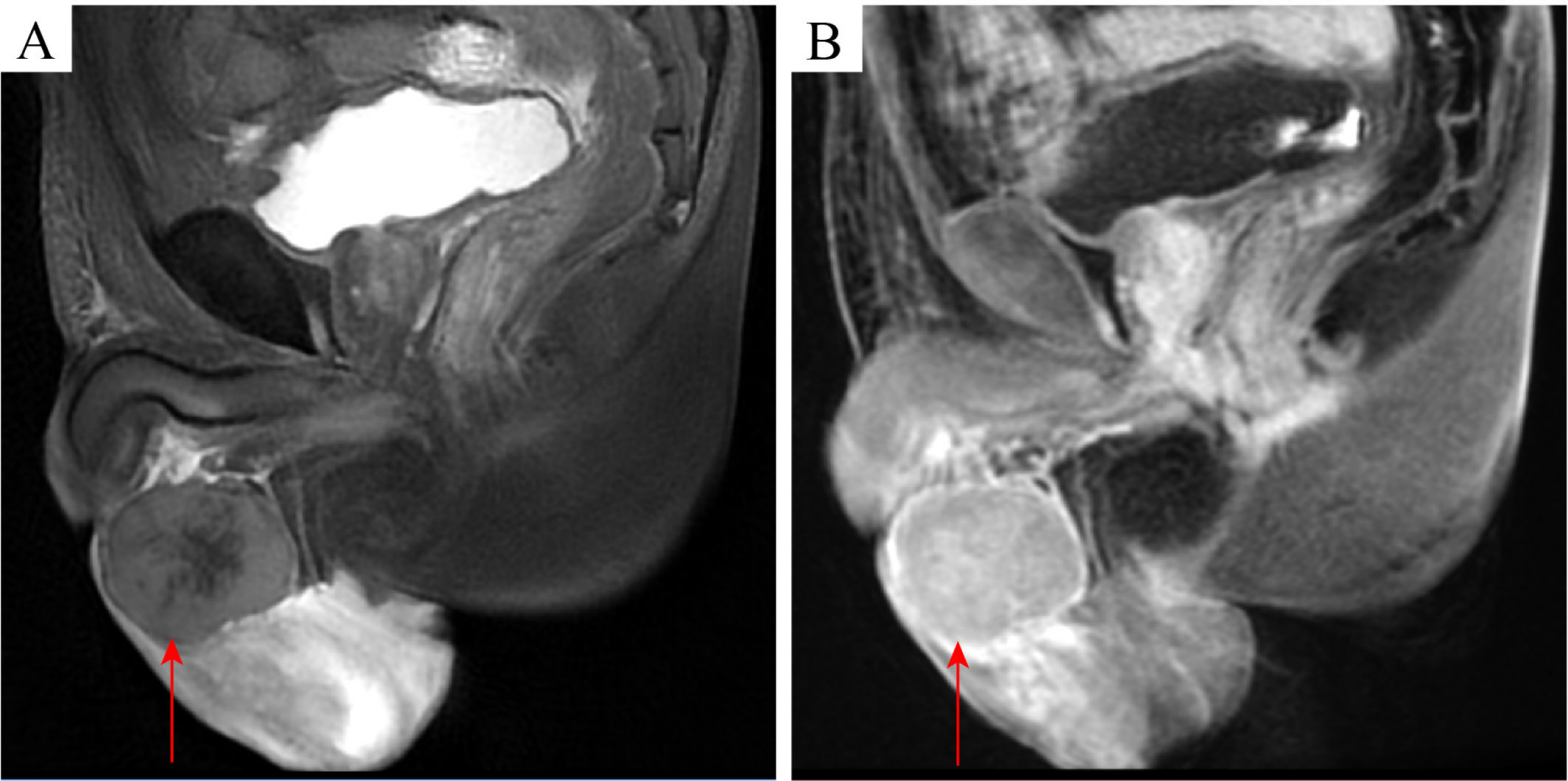
Pelvic MRI of the left scrotal mass. **(A)** T2-weighted image demonstrates a heterogeneous cystic-solid mass (52 mm × 56 mm × 50 mm) in the left epididymal region (arrow), with irregular margins and mixed signal intensity. **(B)** Contrast-enhanced T1-weighted image reveals marked peripheral rim enhancement of the mass (arrow), with unclear boundaries between the inferior margin of the mass and the left testis.

**Figure 2:**
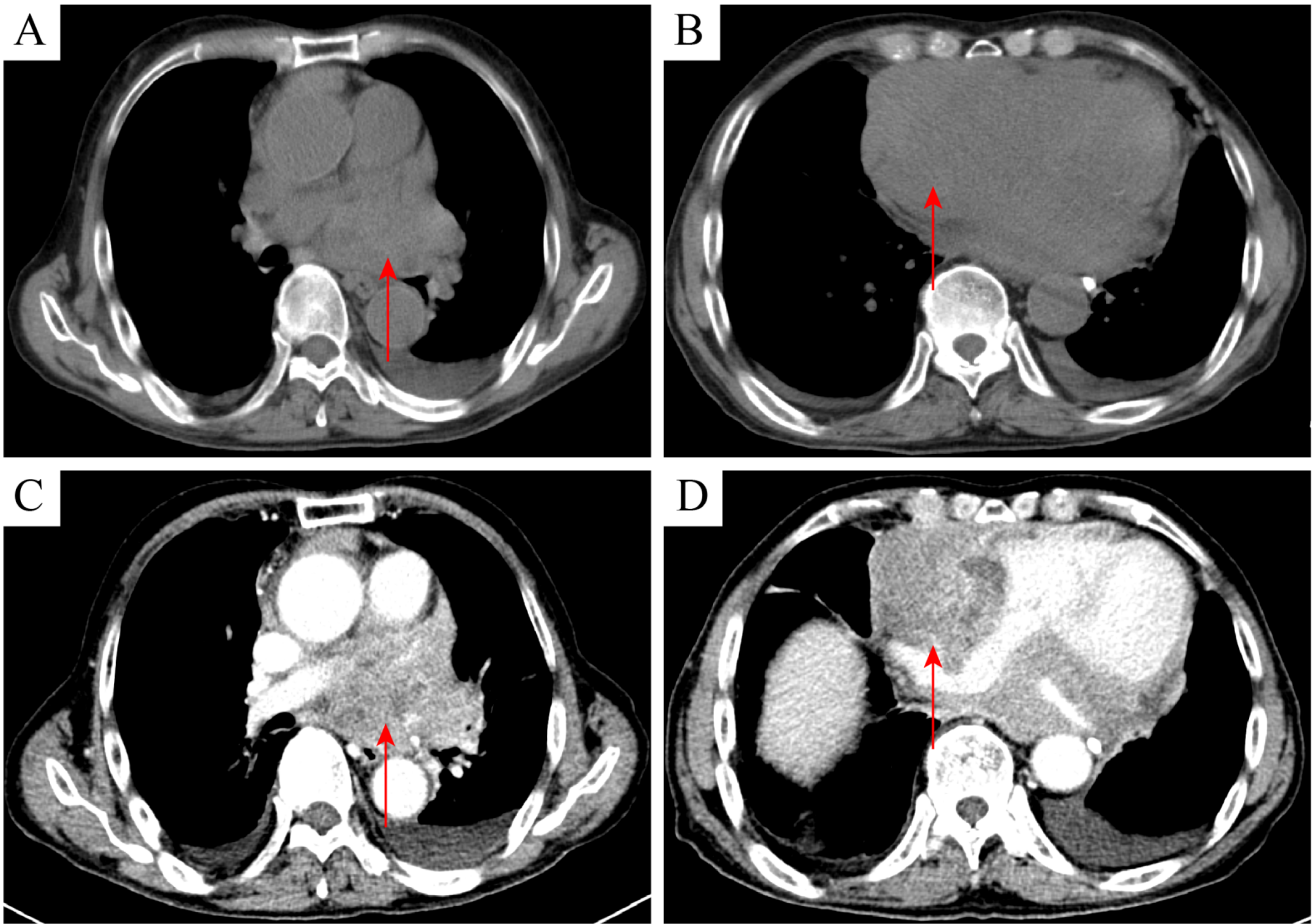
Chest CT plain scan + enhancement demonstrating mediastinal lesions. **(A, B)** Plain scan of chest CT showed an irregular soft tissue mass in the right anterior mediastinum, middle mediastinum, and posterior mediastinum (arrow), with uneven density and unclear demarcation from the normal mediastinal structure. **(C, D)** Chest CT enhancement showed uneven enhancement of the mass, narrowed compression of adjacent blood vessels and cardiac cavity, and undersmooth margins.

**Figure 3:**
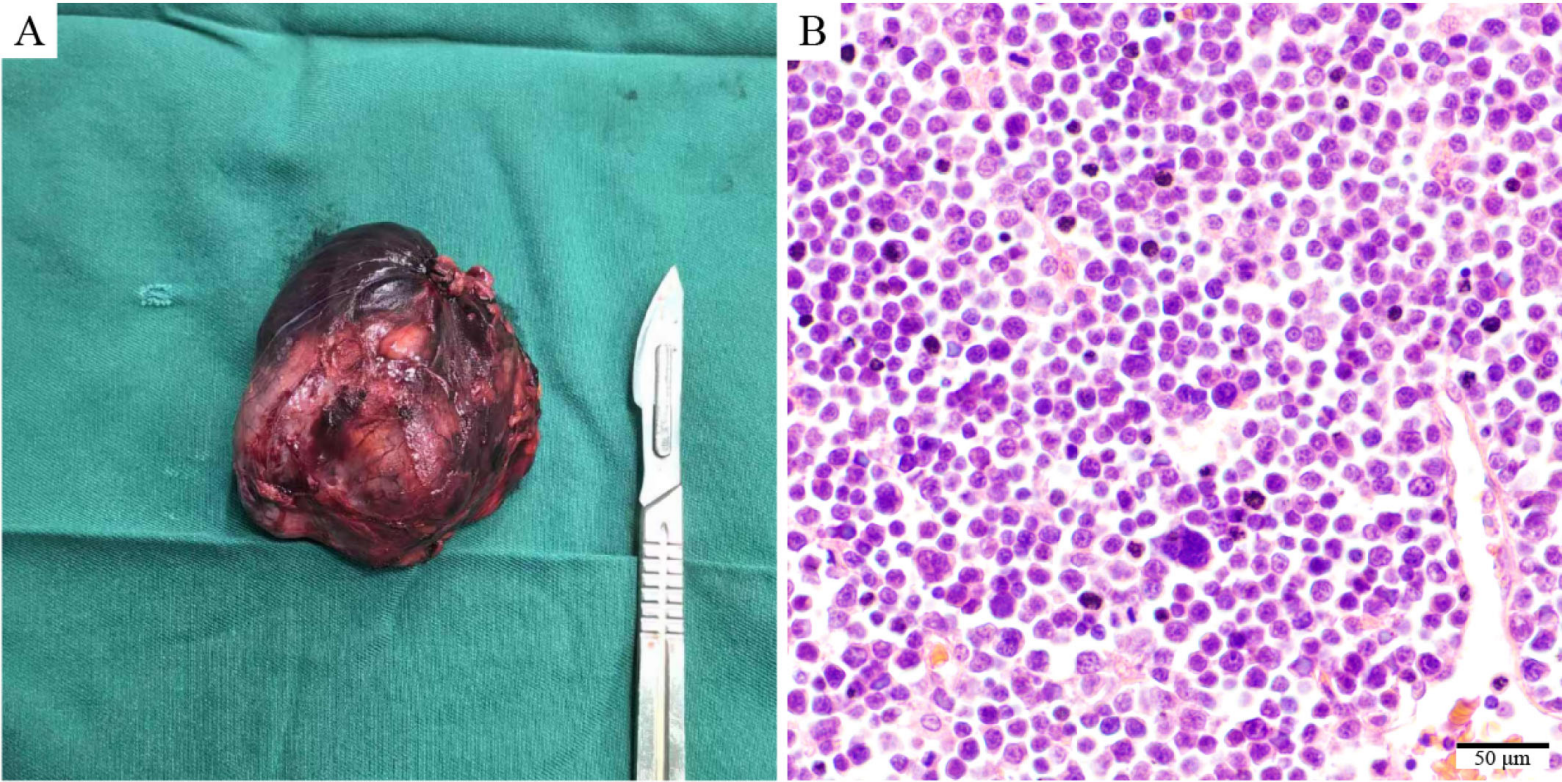
Gross and histopathological features of the left scrotal mass. **(A)** Gross specimen of the resected left scrotal mass (70 mm × 70 mm × 60 mm), showing focal areas of necrosis. **(B)** Hematoxylin and eosin (H&E) staining (×200, scale bar: 50 μm) reveals sheets of neoplastic cells with round to oval nuclei, prominent nucleoli, and high nuclear-to-cytoplasmic ratio.

**Figure 4:**
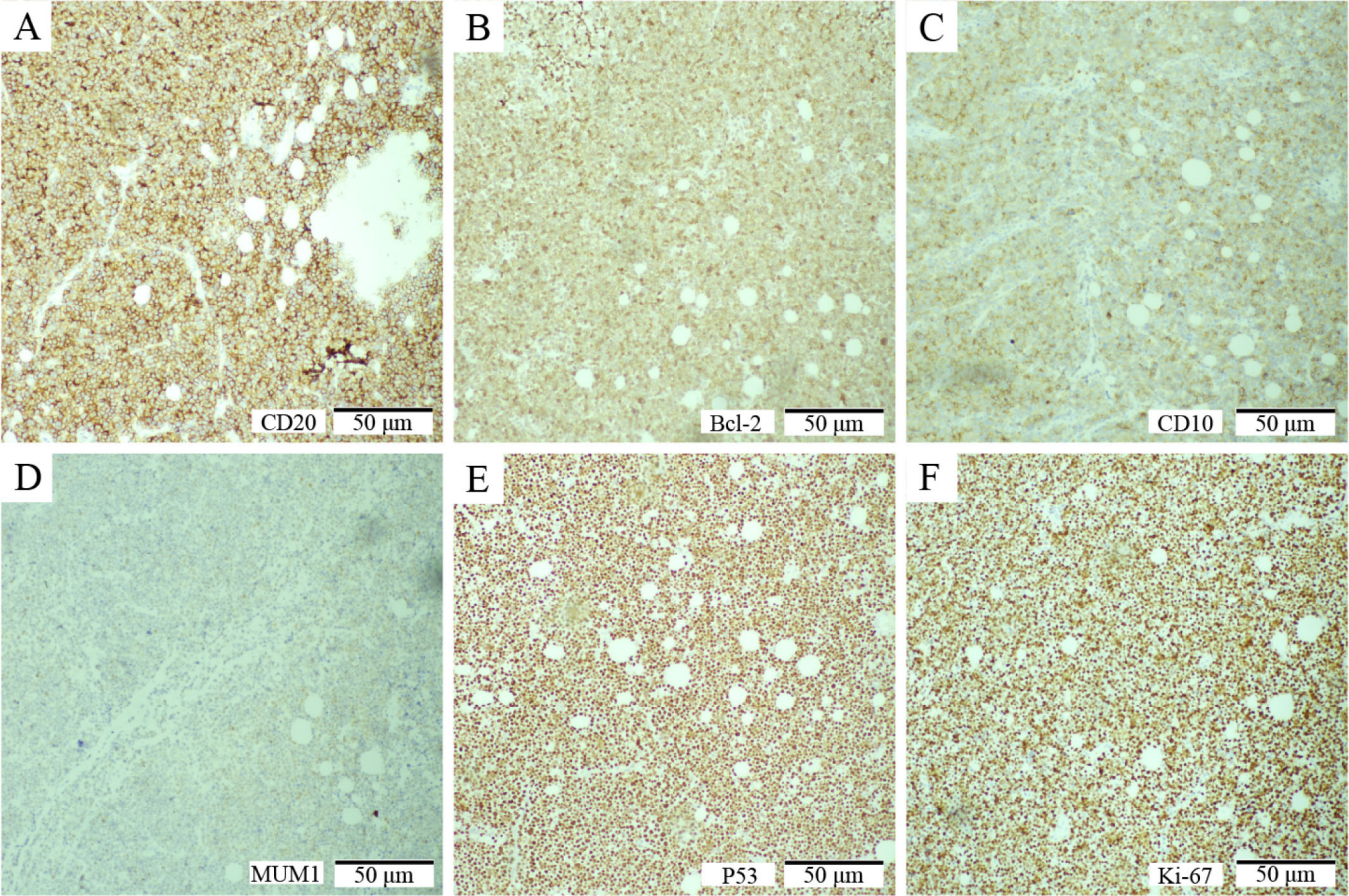
IHC features of the left scrotal diffuse large B-cell lymphoma (DLBCL). **(A)** CD20 immunostaining (×200, scale bar: 50 μm) demonstrates (++), confirming B-cell lineage. **(B)** Bcl-2 expression (×200, scale bar: 50 μm) is (++), indicating anti-apoptotic activity. **(C)** CD10 staining (×200, scale bar: 50 μm) shows (+), supporting a germinal center B-cell-like (GCB) subtype. **(D)** MUM1 immunoreactivity (×200, scale bar: 50 μm) is (+), consistent with the GCB phenotype. **(E)** P53 protein overexpression (×200, scale bar: 50 μm) with a missense mutation pattern (+), suggesting TP53 dysregulation. **(F)** Ki-67 index (×200, scale bar: 50 μm) exceeds 90% in hotspot areas, reflecting high tumor proliferative activity.

An 81-year-old male patient visited the Affiliated Hospital of Zunyi Medical University in February 2024 for a progressive enlargement of a palpable left scrotal mass over a month. His medical history included primary hypertension diagnosed 20 years prior, cerebral infarction diagnosed 10 years prior, and high-grade urothelial carcinoma of the bladder diagnosed 5 years prior. There was no history of diabetes, familial genetic disorders, or trauma. The patient has recently experienced weakness in both lower limbs but denied fever, night sweats, cough, chest pain, abdominal pain, hematuria, or weight loss. Physical examination showed an enlarged, non-tender left scrotum with intact local skin and no ulceration or pus. A mass, approximately 50 mm × 40 mm in size, was palpable above the left testis, with an unclear boundary with the testis, no tenderness, hard texture, adhesion to the surrounding skin, poor mobility, and a negative scrotal transillumination test. No abnormalities were observed in the contralateral side. The relevant imaging examinations were completed after admission. Testicular contrast-enhanced ultrasound suggested that a hypoechoic mass measuring 50 mm × 34 mm was found in the left scrotum wall. After injection of the ultrasound contrast agent, the mass showed uneven hyperenhancement in the arterial phase and hyperenhancement in the parenchymal phase. The perfusion of the mass was earlier than that of the normal tissue, and the regression was later than that of the normal tissue. The boundary of the mass was unclear, the shape was irregular, and it was connected with the sheath of the scrotum wall. The possibility of leiomyoma was considered, and the stromal tumor could not be excluded. The pelvic MRI (non-contrast and contrast-enhanced) revealed a cystic-solid mass measuring approximately 52 mm × 56 mm × 50 mm in the left epididymis region, with heterogeneous signal intensity. The enhancement showed non-uniform solid enhancement, accompanied by distinct annular margins. The lower border of the mass was indistinct from the left testis, suggesting possible sarcoma or seminoma (**Figures 1A, B**). Chest CT plain scan + enhancement suggested space-occupying lesions in the right anterior, middle, and posterior mediastinum, with the possibility of metastasis (**Figures 2A–D**). Preoperative serum lactate dehydrogenase (LDH) was elevated at 1,344 U/L (normal: 140–271 U/L), increasing to 2,076 U/L postoperatively. β_2_-Microglobulin was elevated at 5,419 ng/mL (normal: 604–2,286 ng/mL). Serum alpha-fetoprotein (AFP), carcinoembryonic antigen (CEA), carbohydrate antigen 19-9 (CA19-9), CA125, and human chorionic gonadotropin (hCG) levels were within normal limits. Human immunodeficiency virus (HIV), hepatitis B virus (HBV), and hepatitis C virus (HCV) serologies were negative. The initial clinical diagnosis was scrotal sarcoma. To determine the tumor’s nature, a left scrotal mass excision was performed under general anesthesia with the patient’s and family’s consent. During surgery, the mass was located above the left testicle, presenting as a firm, poorly mobile mass with dense adhesions to surrounding tissues. The mass extended to the base of the penis. Given the preserved boundary between the mass and testicle, the mass was completely removed while preserving the left testicle. The postoperative pathological examination was based on this excised specimen. The specimen measured approximately 70 mm × 70 mm × 60 mm, with a gray-brown cut surface showing localized necrosis (**Figure 3A**). Histopathological examination of the resected specimen revealed a neoplastic lesion composed of tumor cells with round to oval morphology, characterized by enlarged nuclei and prominent nucleoli (**Figure 3B**). IHC staining demonstrated the following profile: *CD20* (++), *Bcl-2* (++), *CD10* (+), *MUM1* (+), *BCL-6* (−), *P53* (aberrant overexpression, suggestive of missense mutation), *CD117* (−), *CD21* (−), *CD3* (−), *OCT3/4* (−), *PLAP* (−), *anti-myeloid leukemia 4* (*AML4*) (−), *vimentin* (−), *c-Myc* (−), cyclin D1 (−), and *Ki-67* (>90% in hotspot regions) (**Figures 4A–F**).

The patient was ultimately diagnosed with left scrotal DLBCL (GCB subtype). To further exclude high-grade B-cell lymphomas (such as double-/triple-hit lymphomas), FISH testing was performed. The results showed negative *MYC* and *BCL-2* gene rearrangements, thereby ruling out double-hit lymphoma. According to the Ann Arbor lymphoma staging system, the patient was classified as stage IV, with an International Prognostic Index (IPI) score of 5 (high-risk) and an Eastern Cooperative Oncology Group (ECOG) score of 2. After confirming the diagnosis and recommending R-CHOP chemotherapy, we conducted a comprehensive evaluation of the patient. Given the patient’s advanced age, multiple comorbidities (hypertension, history of cerebral infarction), significant concerns about chemotherapy toxicity, and personal/family preference for palliative care, the patient and family ultimately declined all chemotherapy options following thorough discussion. The patient passed away 3 months after diagnosis due to rapid disease progression.

## Discussion

DLBCL is the most common NHL, with approximately 30% of cases originating extranodally (3). In the male urogenital system, primary testicular lymphoma is relatively common (4), while PS-DLBCL is extremely rare. Both this case report and previous studies (**Table 1**) indicate that PS-DLBCL is characterized by aggressive invasiveness, high misdiagnosis rates, and poor prognosis. PS-DLBCL typically presents as a painless, firm scrotal mass, with or without a dragging sensation, and may develop systemic symptoms in advanced stages (5). The differential diagnosis requires comprehensive evaluation, encompassing a wide range of conditions from benign lesions (e.g., hydrocele, epididymitis) to malignant tumors (e.g., seminoma, soft tissue sarcoma, squamous cell carcinoma, and metastatic cancer).

**Table 1:** Comparative analysis of scrotal lymphomas and related malignancies.

Author (year)	Age (years)	Primary site	Presenting symptom(s)	Clinical/radiologic findings	Histopathological diagnosis	Diagnostic workup	Treatment	Outcome	Clinical significance
Xu et al. (29) (2011)	53	Scrotal skin	Multiple scrotal nodules and plaques for 6 months	Ulcerated nodules, inguinal lymphadenopathy	CD8^+^ primary cutaneous anaplastic large cell lymphoma	IHC+: CD30, CD8, CD3 IHC−: ALK Elevated LDH CT: skin thickening	4 cycles of CHOP chemotherapy	Complete remission; on surveillance	Rare CD8^+^ phenotype in the genital region
Chung et al. (30) (2007)	65	Scrotal skin	Foul-smelling scrotal mass for >2 years	Verrucous carcinoma, ulceration, massive inguinal lymphadenopathy	Verrucous carcinoma and synchronous peripheral T-cell lymphoma	HPV 16/18+; IHC+ (LCA, CD4); CT: widespread lymphadenopathy	Wide local excision + inguinal lymph node dissection	Died of sepsis 2 months postoperatively	First reported synchronous VC and PTCL
Our case (2024)	81	Scrotal wall	Progressively enlarging left scrotal mass for 1 month	Hard mass; mediastinal involvement	DLBCL, GCB subtype	IHC+: CD20, BCL-2; Ki-67 >90%; FISH: MYC/BCL-2 negative; LDH↑↑, β_2_-MG↑↑; CT/MRI: mass and metastases	Surgical excision only (declined systemic therapy)	Rapid disease progression; died 2 months postoperatively	Primary scrotal DLBCL; ultra-high proliferative index (Ki-67 > 90%)

β2-MG, beta-2 microglobulin; BCL-2, B-cell lymphoma 2; CHOP, cyclophosphamide, doxorubicin, vincristine, prednisone; CT, computed tomography; DLBCL, diffuse large B-cell lymphoma; FISH, fluorescence *in situ* hybridization; GCB, germinal center B-cell-like; HPV, human papillomavirus; IHC, immunohistochemistry; LDH, lactate dehydrogenase; MRI, magnetic resonance imaging; PTCL, peripheral T-cell lymphoma.

In terms of auxiliary examination, ultrasound is the preferred imaging method for the evaluation of scrotal masses, and its main role is to distinguish between malignant lesions in the testis and benign lesions outside the testis (6). On ultrasound, PS-DLBCL typically appears as a homogeneous hypoechoic mass with abundant internal blood flow. CT is generally not used for initial evaluation but rather for staging and follow-up of malignant tumors (including lymphoma) (6). MRI demonstrates significant advantages in distinguishing malignant from benign testicular lesions, with 100% sensitivity and 87.5% specificity in histopathological comparison studies (7, 8). ^18^F-FDG PET/CT is pivotal in the diagnosis and treatment of DLBCL. Unlike conventional contrast-enhanced CT, PET/CT provides both metabolic and anatomical data, enabling more sensitive detection of extranodal involvement (e.g., spleen, bone marrow) and reducing the risk of underestimating disease stages (9, 10). Approximately 5.4% of patients may consequently receive a higher clinical stage classification (10). This technology serves not only as the gold standard for staging and tumor burden assessment in Ann Arbor but also as a critical basis for efficacy evaluation (using the Deauville criteria) and prognosis prediction. It has become the core imaging method for DLBCL risk stratification, treatment decision-making, and central nervous system (CNS) prevention strategy formulation (9, 10). Furthermore, serum tumor markers such as hCG, AFP, and LDH help exclude germ cell tumors (7). Notably, LDH levels serve as a key indicator of tumor burden, invasion potential, and prognosis, while also predicting CNS involvement in NHL patients (11).

The definitive diagnosis of PS-DLBCL relies on histopathological and IHC analysis. The tumor features characteristic patterns of DLBCL, presenting with prominent nucleoli and proliferating cells (12). IHC demonstrates B-cell marker expression (e.g., CD20, CD79a) in tumor cells (12). In this case, the Ki-67 proliferation index was abnormally elevated (>90%) with a P53 frameshift mutation, indicating aggressive disease (3). FISH can detect clinically significant genetic rearrangements. High-grade B-cell lymphomas with MYC and BCL-2 and/or BCL-6 rearrangements occur in 5% to 10% of DLBCL cases, commonly referred to as double- or triple-hit lymphomas (13). This is closely associated with a poor prognosis. In this case, Ki-67 expression was over 90%. To confirm the presence of MYC and BCL-2 rearrangements in the high-grade B-cell lymphoma, we performed FISH for myc and Bcl-2 genes, which yielded negative results, confirming that the patient did not have high-grade B-cell lymphoma.

The “skip metastasis” observed in this PS-DLBCL case may be the result of multiple factors working together. First, the formation of immune-privileged sites may facilitate tumor cell colonization in the mediastinum. Studies have demonstrated that tumor-draining lymph nodes (TDLNs) can be reprogrammed into a highly immunosuppressive microenvironment through tumor-derived signals (e.g., PD-L1-exocytosed exosomes). This transformation manifests as a massive accumulation of immunosuppressive cells such as regulatory T cells (Tregs) and regulatory dendritic cells (mregDCs), along with upregulation of immune checkpoint molecules like PD-1/PD-L1. These coordinated mechanisms compromise the immune surveillance function of local CD8^+^ T cells, enabling mediastinal lymph nodes to serve as “sanctuaries” where tumor cells evade immune attack and establish successful colonization (14–16). Secondly, hematogenous dissemination provides a direct pathway to bypass regional lymph nodes. Evidence suggests that tumor cells can invade blood vessels within lymph nodes, subsequently entering the systemic circulation to achieve distant organ metastasis. This mechanism may explain why tumor cells can completely bypass the inguinal to retroperitoneal lymphatic chains and directly reach the blood-rich mediastinal region (17). Finally, abnormal lymphatic drainage forms the structural basis for this skip metastasis: By secreting lymphangiogenic factors like VEGF-C, the tumor induces lymphangiogenesis and altered drainage pathways in TDLNs. This tumor-driven remodeling of the lymphatic network may redirect lymphatic flow, enabling primary scrotal tumor cells to directly “skip” to mediastinal lymph nodes through abnormally established lymphatic channels (18–20). Therefore, the formation of immune-privileged sites, the use of hematogenous pathways, and the abnormal changes in lymphatic structure may collectively constitute the potential biological basis for the rare skip metastasis in this case.

DLBCL is a curable disease. For years, the 6–8-cycle R-CHOP regimen (rituximab, cyclophosphamide, doxorubicin, vincristine, prednisone) has been the standard first-line treatment for DLBCL, achieving a cure rate of approximately 60%–70% (9, 21). PS-DLBCL is a rare disease. However, due to its superficial location, it can be completely removed through surgery if detected early, thereby achieving the purpose of diagnosis and treatment. In a phase II clinical trial conducted by Nowakowski et al. (22), it was indicated that lenalidomide combined with the R-CHOP regimen as the first-line treatment could increase the remission rate and improve survival in patients with newly diagnosed DLBCL. Furthermore, the latest study by Tilly et al. (23) demonstrated that in previously untreated intermediate- or high-risk DLBCL patients, pola-R-CHP (polatuzumab vedotin in combination with rituximab and cyclophosphamide) showed a lower risk of disease progression, recurrence, or death compared to those receiving R-CHOP (rituximab, cyclophosphamide, and prednisone).

In addition to selecting an appropriate systemic regimen, CNS prophylaxis is a critical consideration in high-risk DLBCL patients, such as the one presented here. For this DLBCL patient, CNS prophylaxis is a critical clinical consideration. Although CNS recurrence in DLBCL is uncommon (approximately 2%–5%), its occurrence is associated with a poor prognosis (24). The imaging findings may present as atypical non-enhancing infiltrative lesions, which can lead to diagnostic delays (24). The established high-risk factors include involvement of specific extra-lymphatic sites such as the testes and breast, double-hit/double-expressing lymphoma, advanced disease, and high IPI scores (25–27). This patient exhibits all three critical features: primary scrotal cancer, stage IV progression, and a high IPI score, placing him in the high-risk category for CNS recurrence. Therefore, when initiating treatment, clinicians should strongly recommend CNS prophylaxis strategies—such as intrathecal methotrexate or high-dose systemic methotrexate—alongside initial systemic chemotherapy to reduce the risk of this life-threatening complication. However, the patient refused all subsequent treatment for personal reasons, highlighting the importance of adequate communication and shared decision-making about the benefits and risks of treatment in older, high-risk patients.

The poor prognosis of this case is associated with multiple high-risk factors. First, the patient was diagnosed at an advanced stage (Ann Arbor stage IV) with an IPI score of 5, placing him in the high-risk group (28). Second, the tumor exhibited highly aggressive biological behavior, specifically: Ki-67 expression exceeded 90% in hotspot regions, typically associated with rapid tumor growth and poor prognosis (3); IHC revealed P53 protein overexpression (frameshift mutation pattern), indicating TP53 functional inactivation—a known independent prognostic marker for DLBCL (3); GCB subtype: although GCB subtypes are generally considered more favorable than non-GCB subtypes, their prognosis remains concerning when combined with other high-risk factors (such as advanced stage, high Ki-67, and TP53 alterations in this case) (3). During treatment planning, we assessed the patient’s physical status with an ECOG score of 2. This indicates the patient can perform self-care but cannot engage in work activities and can get out of bed for activities over 50% of the day. This compromised performance status was also a key factor influencing the treatment decision.

In summary, PS-DLBCL is a rare extranodal lymphoma typically presenting as a progressive, painless scrotal mass in elderly men. Due to its atypical clinical manifestations and late-stage diagnosis, early detection, treatment, and prognosis of this disease face significant challenges. Early biopsy or surgical resection to obtain definitive histopathological and IHC diagnoses is crucial. After confirmation, multidisciplinary collaboration among urologists, hematologic oncologists, radiologists, and pathologists is essential to develop optimal staging protocols and personalized treatment plans. This article describes the diagnosis, pathology, treatment, and prognosis of PS-DLBCL to enhance clinicians’ understanding and systematic management.

## Data Availability

The original contributions presented in the study are included in the article/supplementary material. Further inquiries can be directed to the corresponding author.
